# Neoadjuvant chemoradiotherapy plus programmed cell death protein-1 blockade versus chemoradiotherapy alone for muscle-invasive bladder cancer: a real-world comparative study

**DOI:** 10.3389/fimmu.2025.1711690

**Published:** 2025-11-28

**Authors:** Fan Zhang, Jingsuo Wang, Yanhang Yu, Chuanao Zhang, Jun Ouyang, Zhiyu Zhang

**Affiliations:** 1 Department of Urology, First Affiliated Hospital of Soochow University, Suzhou, China; 2 Department of Nephrology, First Affiliated Hospital of Soochow University, Suzhou, China

**Keywords:** muscle-invasive bladder cancer, neoadjuvant chemoradiotherapy, PD-1 inhibitor, pathological complete response, safety

## Abstract

**Introduction:**

Muscle-invasive bladder cancer (MIBC) has a high risk of recurrence and mortality despite radical cystectomy. Neoadjuvant chemoradiotherapy (NACRT) offers has been investigated as a bladder-preserving strategy in selected patients; however, in this study, all patients underwent radical cystectomy following NACRT, and programmed cell death protein-1 (PD-1) inhibitors have shown antitumor activity in urothelial carcinoma. Combining NACRT with PD-1 blockade may enhance tumor response; however, its pathological benefits and short-term safety remain unclear. This study aimed to compare pathological response and short-term safety between NACRT alone and NACRT combined with PD-1 blockade (NACRT + PD-1) in patients with MIBC.

**Methods:**

A retrospective review was conducted for 59 consecutive patients with MIBC (cT2–T4aN0M0) treated at the First Affiliated Hospital of Soochow University between January 2021 and December 2024. Twenty-seven patients received NACRT alone, and 32 received NACRT plus a PD-1 inhibitor—toripalimab (n=18) or tislelizumab (n=14). Chemotherapy comprised gemcitabine 1,000 mg/m² (days 1 and 8) and cisplatin 70 mg/m² (days 2–3) every 21 days for ≥3 cycles. Intensity-modulated pelvic radiotherapy delivered 45 Gy in 25 fractions plus a weekly boost (10 Gy in five fractions) to the bladder. The PD-1 antibody (toripalimab 200 mg or tislelizumab 240 mg) was infused on day 8 of each cycle. The primary endpoints were pathological downstaging rate (PDR, ≤ypT1/Tis/TaN0M0) and pathological complete response rate (PCRR, ypT0N0M0). The secondary endpoint was treatment-related adverse events (AEs) graded using the Common Terminology Criteria for Adverse Events version 5.0. Group comparisons used χ², Fisher’s exact, or non-parametric tests as appropriate (two-sided, α=0.05).

**Results:**

Baseline demographics and clinical characteristics were balanced between the groups (all p > 0.05). After radical cystectomy, pathological stage distribution did not differ significantly (p > 0.05). The PDR was 74.07% (20/27) in the NACRT group and 87.50% (28/32) in the NACRT + PD-1 group (p=0.187). The PCRR increased from 44.44% (12/27) with NACRT alone to 71.88% (23/32) with PD-1 addition (p=0.033). Toripalimab and tislelizumab achieved comparable PDRs (83.33% *vs*. 92.86%, p=0.613) and PCRRs (66.67% *vs*. 78.57%, p=0.694). No grade ≥4 AEs or treatment-related deaths occurred, and AE frequencies were similar between groups (all p > 0.05).

**Conclusion:**

NACRT combined with PD-1 blockade significantly improved PCRR without increasing toxicity in patients with MIBC. These findings support conducting a prospective single-arm phase II multicenter trial to confirm potential long-term survival benefits.

## 1 Introduction

Bladder cancer is the 10^th^ most common malignancy worldwide, with an estimated 573,000 new cases and 213,000 deaths annually (1). Approximately one-third of cases present as muscle-invasive bladder cancer (MIBC), a biologically aggressive subtype with a 5-year overall survival rate of 50% despite aggressive local treatment (2). Cisplatin-based neoadjuvant chemotherapy (NAC) followed by radical cystectomy (RC) remains the standard of care; however, only 30–40% of patients achieve meaningful tumor regression, and more than 40% experience recurrence within 3 years (3–5).

Neoadjuvant chemoradiotherapy (NACRT) has emerged as an alternative cytoreductive strategy that may improve local control while preserving peri-tumoral vasculature; thereby, enhancing drug delivery (6, 7). Immune checkpoint inhibitors (ICIs), particularly programmed cell death protein-1 (PD-1) and programmed death ligand-1 (PD-L1) antibodies, have transformed the treatment of advanced urothelial carcinoma (8–10). Both radiotherapy and certain cytotoxic agents can promote immunogenic cell death, upregulate tumor antigen presentation, and increase PD-L1 expression, providing a strong biological rationale for combining NACRT with PD-1 blockade (11, 12).

The clinical benefit of combining NACRT with PD-1 inhibition in the neoadjuvant treatment of MIBC remains uncertain. Therefore, this single-center retrospective cohort study aimed to compare the pathologic downstaging rate (PDR), pathological complete response rate (PCRR), and acute treatment-related toxicity between NACRT alone and NACRT plus PD-1 inhibition.

## 2 Materials and methods

### 2.1 Study design and patients

**Figure 1:**
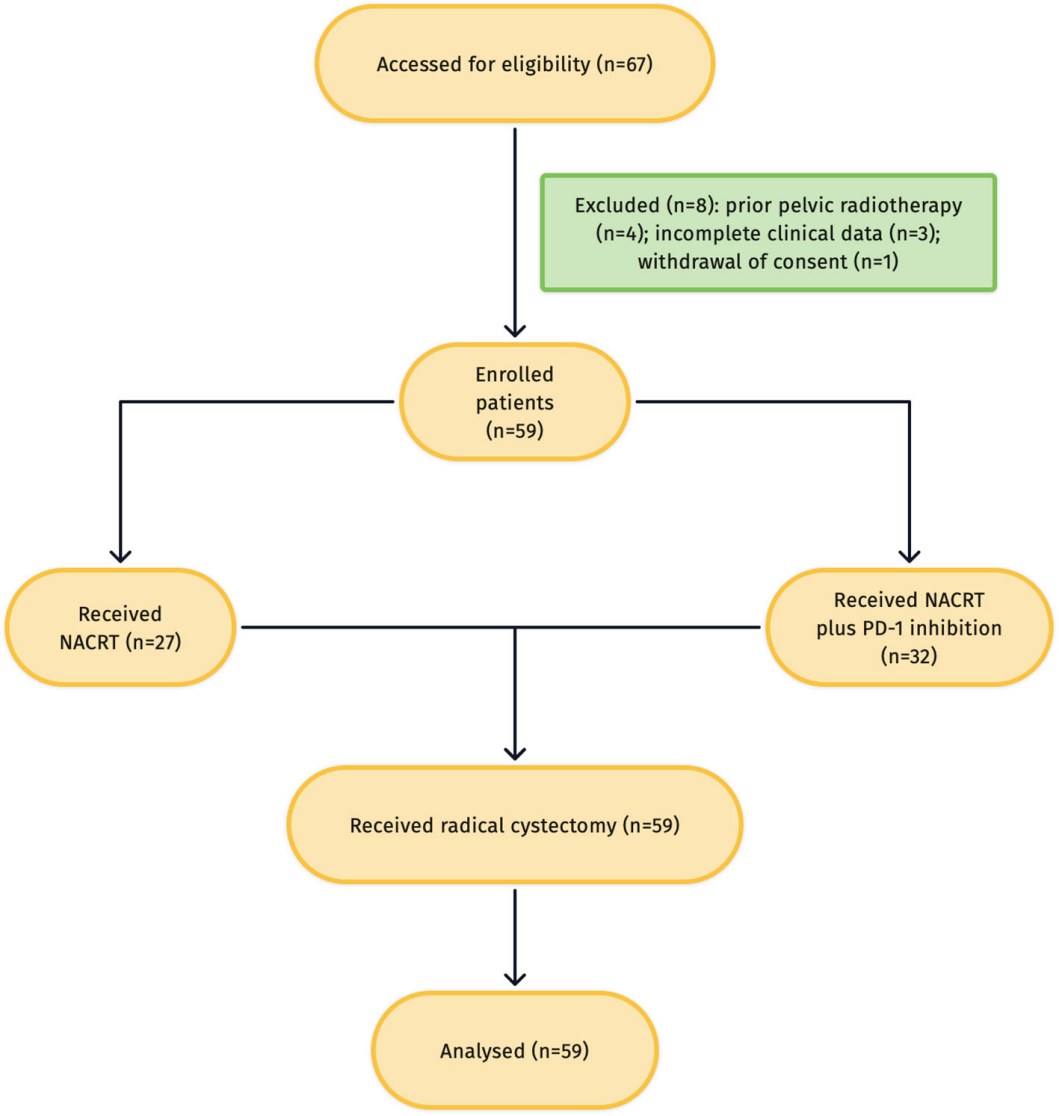
Flow chart of patient selection and exclusion for the study cohort.

All consecutive patients with MIBC who underwent neoadjuvant therapy followed by RC at the First Affiliated Hospital of Soochow University between 1 January 2021 and 31 December 2024 were screened for eligibility. Inclusion criteria were: (i) histologically confirmed urothelial carcinoma with muscle invasion on initial transurethral resection of the bladder tumor (TURBT); (ii) clinical stage cT2–T4aN0M0; (iii) receipt of ≥3 cycles of neoadjuvant therapy; and (iv) availability of complete perioperative records. Exclusion criteria were: other active malignancies, active infection, distant metastasis, refusal to undergo RC, or incomplete clinical data. The flow chart of patient selection and exclusion for the study cohort was shown in **Figure 1**. This study was approved by the Ethics Committee of the First Affiliated Hospital of Soochow University (approval number: 577, 2024), and written informed consent was obtained from all participants.

### 2.2 Treatment regimens

Patients in the NACRT group received intensity-modulated pelvic radiotherapy at a total dose of 45 Gy in 25 fractions, plus a weekly boost of 10 Gy in five fractions directed to the bladder. Radiotherapy was administered concurrently with chemotherapy consisting of gemcitabine 1000 mg/m² on days 1 and 8, and cisplatin 70 mg/m² on day 2 of each 21-day cycle, for three cycles (10, 13).

Patients in the NACRT plus PD-1 blockade group received the same NACRT regimen, with the addition of either toripalimab 200 mg or tislelizumab 240 mg administered intravenously on day 8 of each cycle (10).

Laboratory monitoring, including complete blood count, serum chemistry, thyroid function tests, myocardial enzyme profile, and cortisol levels, was performed prior to every treatment cycle. Radiologic restaging with multiparametric magnetic resonance imaging (mpMRI) or contrast-enhanced computed tomography (CT) was conducted within 2 weeks before RC.

### 2.3 Endpoints

The primary endpoints were:

- PDR: defined as a final cystectomy pathology stage of ≤ypT1/Tis/TaN0M0 (10).
- PCRR: defined as ypT0N0M0 on final cystectomy pathology.

The secondary endpoint was the incidence and grade of treatment-related adverse events (AEs) from the first treatment dose to the preoperative assessment, graded according to the Common Terminology Criteria for Adverse Events (CTCAE) version 5.0 (14).

### 2.4 Sample size calculation

*A priori* power analysis was performed using PASS version 15.0 (NCSS, Kaysville, UT, USA). Assuming that the PCRR would improve from 45% with NACRT alone (based on previously reported data (10)) to 70% with NACRT plus PD-1 blockade (hypothesized based on the strong biological rationale for combining chemoradiotherapy with immunotherapy and early clinical signals from neoadjuvant chemo-immunotherapy trials, representing an absolute difference of 25%), a two-sided χ² test with α=0.05 and β=0.20 (power=80%) indicated that a minimum of 120 participants (60 per arm, allocation ratio 1:1) was required.

### 2.5 Statistical analysis

Statistical analyses were conducted using R (version 4.2.1), employing functions from the “stats” (v4.2.1), “rms” (v6.4.0), “pwr(v1.3-0)” and “ResourceSelection” (v0.3-5) packages. Continuous variables were summarized as mean ± standard deviation (SD) and compared between groups using Student’s t-test when both normality and homoscedasticity assumptions were satisfied. When variances were unequal, Welch’s t-test was applied, and in cases where the normality assumption was violated, the Wilcoxon rank−sum test was used. Categorical variables were expressed as counts (percentages) and compared using Pearson’s χ² test when all expected cell frequencies exceeded 5 and the total sample size was ≥40. Pearson’s χ² test with Yates’ continuity correction was applied when expected frequencies ranged from 1 to 5. Fisher’s exact test was used for cases in which any expected frequency was <1 or the overall sample size was <40. Univariate and multivariate logistic regression models were constructed to identify independent predictors of PCRR. All statistical tests were two minus;sided, and values of p < 0.05 were considered statistically significant.

## 3 Results

### 3.1 Baseline demographic and clinical characteristics

Among the 59 eligible patients, 27 received NACRT alone, whereas 32 underwent NACRT plus PD-1 inhibition. Notably, all patients completed at least three cycles of neoadjuvant therapy without any dose reduction. The two cohorts were well balanced at baseline. The mean age was 66.56 ± 9.81 years in the NACRT alone group and 67.06 ± 10.24 years in the NACRT plus PD-1 inhibition group. The proportion of male patients was 66.67% and 75.00%, respectively. No statistically significant differences were observed in body mass index, high blood pressure, diabetes mellitus, hyperlipidemia, smoking history, Eastern Cooperative Oncology Group performance status, Charlson Comorbidity Index, histological grade, or clinical T stage (all p > 0.05; **Table 1**).

**Table 1:** Baseline demographic and clinical characteristics of patients with MIBC treated with NACRT alone versus NACRT + PD-1 blockade.

Characteristics	NACRT + PD-1	NACRT	P value
n	32	27	
Sex, n (%)			0.481
Male	24 (75.00%)	18 (66.67%)	
Female	8 (25.00%)	9 (33.63%)	
Age (years), mean ± SD	67.06 ± 10.24	66.56 ± 9.81	0.848
BMI (kg/m^2^), mean ± SD	23.60 ± 3.85	23.60 ± 3.56	0.998
HBP, n (%)			0.703
Yes	17 (53.13%)	13 (48.15%)	
No	15 (46.88%)	14 (51.85%)	
DM, n (%)			0.550
Yes	8 (25.00%)	5 (18.52%)	
No	24 (75.00%)	22 (81.48%)	
HLP, n (%)			0.448
Yes	10 (31.25%)	11 (40.74%)	
No	22 (68.75%)	16 (59.26%)	
Smoking history, n (%)			0.636
Yes	15 (46.88%)	11 (40.74%)	
No	17 (53.13%)	16 (59.26%)	
ECOG score, n (%)			0.199
0	13 (40.63%)	14 (51.85%)	
1	8 (25.00%)	10 (37.04%)	
2	9 (28.13%)	2 (7.41%)	
3	2 (6.25%)	1 (3.70%)	
Charlson Comorbidity Index, mean ± SD	1.03 ± 0.69	1.00 ± 0.92	0.688
Histological grade, n (%)			0.187
High grade	28 (87.50%)	20 (74.07%)	
Low grade	4 (12.50%)	7 (25.93%)	
cT stage, n (%)			0.118
cT2	18 (56.25%)	8 (29.63%)	
cT3	9 (28.13%)	13 (48.15%)	
cT4a	5 (15.63%)	6 (22.22%)	

Histological grade refers to the initial diagnostic TURBT pathology confirming muscle invasion. While low-grade MIBC is rare, these cases were confirmed to have muscle invasion by pathology. BMI, body mass index; HBP, high blood pressure; DM, diabetes mellitus; HLP, hyperlipidemia; ECOG, Eastern Cooperative Oncology Group; NACRT, neoadjuvant chemoradiotherapy; PD-1, programmed cell death protein-1; SD, standard deviation.

### 3.2 Pathological complete response

Post−RC pathology revealed a significantly higher pathological complete response rate (PCRR) in the NACRT plus PD−1 inhibition group compared with NACRT alone (71.88% [23/32] *vs*. 44.44% [12/27], p=0.033; **Table 2**). Partial pathological responses (ypT1/Tis/Ta) were observed in 15.63% and 29.63% of patients, respectively, yielding pathological downstaging rates (PDRs) of 87.50% and 74.07% (p=0.187).

**Table 2:** Pathological downstaging and complete response outcomes after radical cystectomy in patients treated with NACRT alone versus NACRT + PD-1 blockade.

Characteristics	NACRT + PD-1	NACRT	P value
n	32	27	
Pathological outcome, n (%)			0.094
ypT0	23 (71.88%)	12 (44.44%)	
ypT1/Tis/Ta	5 (15.63%)	7 (25.93%)	
≥ypT2	4 (12.50%)	8 (29.63%)	
PDR, n (%)			0.187
Yes	28 (87.50%)	20 (74.07%)	
No	4 (12.50%)	7 (25.93%)	
PCRR, n (%)			0.033
Yes	23 (71.88%)	12 (44.44%)	
No	9 (28.13%)	15 (55.56%)	

PDR, pathological downstaging rate; PCRR, pathological complete response rate; NACRT, neoadjuvant chemoradiotherapy; PD-1, programmed cell death protein-1.

Subgroup analysis by baseline clinical T stage demonstrated that the PCRR advantage with NACRT plus PD−1 blockade was confined to patients with organ−confined disease (cT2), in whom the combination regimen achieved 94.44% (17/18) PCRR versus 50.00% (4/8) with NACRT alone (p=0.020). In contrast, in more advanced tumors (cT3–cT4a), PCRR (42.11% *vs*. 42.86%, p=1.000) and PDR (68.42% *vs*. 71.43%, p=1.000) were comparable between groups (**Table 3**).

**Table 3:** Pathological downstaging and complete response rates stratified by clinical T stage in patients with MIBC.

Characteristics	NACRT+PD1	NACRT	P value
cT2			
n	18	8	
PDR, n (%)			0.308
Yes	18 (100.00%)	7 (87.50%)	
No	0 (0.00%)	1 (12.50%)	
PCR, n (%)			0.020
Yes	17 (94.44%)	4 (50.00%)	
No	1 (5.56%)	4 (50.00%)	
cT3-cT4a			
n	19	14	
PDR, n (%)			1.000
Yes	13 (68.42%)	10 (71.43%)	
No	6 (31.58%)	4 (28.57%)	
PCR, n (%)			1.000
Yes	8 (42.11%)	6 (42.86%)	
No	11 (57.89%)	8 (57.14%)	

To further validate these findings, a multivariate logistic regression model adjusting for clinical T stage, histologic grade, and comorbidity burden (Charlson Comorbidity Index) was constructed (**Table 4**). Clinical T stage remained an independent predictor of PCRR, with cT4a stage significantly reducing the probability of PCR compared with cT2 (OR=17.236, 95% CI=2.703–109.910, p=0.003). Histologic grade and comorbidity burden were not significant in the model.

**Table 4:** Multivariate logistic regression analysis of independent predictors of pathological complete response rate.

Characteristics	Total (N)	Univariate analysis		Multivariate analysis	
		Odds ratio (95% CI)	P value	Odds ratio (95% CI)	P value
cT staging	59				
cT2	26	Reference		Reference	
cT3	22	3.500 (0.967 – 12.672)	0.056	2.767 (0.726 – 10.542)	0.136
cT4a	11	18.900 (3.074 – 116.212)	0.002	17.236 (2.703 – 109.910)	0.003
Histological grade	59				
Low Grade	11	Reference			
High Grade	48	0.786 (0.210 – 2.944)	0.721		
Comorbidity	59				
0	8	Reference			
I	10	0.429 (0.062 – 2.972)	0.391		
II	19	0.462 (0.085 – 2.502)	0.370		
III	22	1.000 (0.198 – 5.045)	1.000		
Treatment regimen	59				
NACRT	27	Reference		Reference	
NACRT+PD1	32	0.313 (0.106 – 0.923)	0.035	0.362 (0.106 – 1.241)	0.106

CI, confidence interval; NACRT, neoadjuvant chemoradiotherapy; PD-1, programmed cell death protein-1.

Taken together, both the stratified and adjusted analyses indicate that the overall PCRR benefit of NACRT plus PD−1 blockade in the entire cohort is primarily driven by the substantial improvement in patients with cT2 disease, while outcomes in cT3–cT4a tumors remain similar between regimens.

### 3.3 Toripalimab versus tislelizumab

Within the NACRT plus PD-1 inhibition group, 18 patients received toripalimab and 14 received tislelizumab. Pathological downstaging occurred in 15/18 (83.33%) and 13/14 (92.86%) patients, respectively (p=0.613). Pathological complete response was achieved in 12/18 (66.67%) of those receiving toripalimab and 11/14 (78.57%) of those receiving tislelizumab (p=0.694) (**Table 5**).

**Table 5:** Pathological downstaging and complete response rates: toripalimab versus tislelizumab within the NACRT + PD-1 cohort.

Characteristics	Toripalimab	Tislelizumab	P value
n	18	14	
Pathological outcome, n (%)			0.854
ypT0	12 (66.67%)	11 (78.57%)	
ypT1/Tis/Ta	3 (16.67%)	2 (14.29%)	
≥ypT2	3 (16.67%)	1 (7.14%)	
PDR, n (%)			0.613
Yes	15 (83.33%)	13 (92.86%)	
No	3 (16.67%)	1 (7.14%)	
PCRR, n (%)			0.694
Yes	12 (66.67%)	11 (78.57%)	
No	6 (33.33%)	3 (21.43%)	

PDR, pathological downstaging rate; PCRR, pathological complete response rate; PD-1, programmed cell death protein-1.

### 3.4 Safety profile

No patient in either treatment arm experienced anaphylaxis, treatment-related deaths, or grade 4 toxicities. AEs such as alopecia, asthenia, appetite or weight loss, nausea, mild transaminase elevation, thrombocytopenia, and rash occurred more frequently in the NACRT plus PD-1 inhibition group compared to the NACRT alone group. Grade 3 events were reported in 16 of 122 events (13.11%) in the NACRT plus PD-1 inhibition group and 10 of 113 events (8.85%) in the NACRT alone group, a difference that was not statistically significant (p=0.298; **Table 6**).

**Table 6:** Incidence and grade of treatment-related adverse events in patients receiving NACRT alone versus NACRT + PD-1 blockade.

Characteristics	NACRT + PD-1	NACRT	P value
n	32	27	
Peripheral sensory neuropathy, n (%)			0.521
0	28 (87.50%)	22 (81.48%)	
1	2 (6.25%)	4 (14.81%)	
2	2 (6.25%)	1 (3.70%)	
Alopecia, n (%)			0.958
0	26 (81.25%)	23 (85.19%)	
1	6 (18.75%)	4 (14.81%)	
Asthenia, n (%)			0.409
0	20 (62.50%)	22 (81.48%)	
1	3 (9.38%)	2 (7.41%)	
2	3 (9.38%)	1 (3.70%)	
3	6 (18.75%)	2 (7.41%)	
Decreased appetite, n (%)			0.256
0	15 (46.88%)	19 (70.37%)	
1	10 (31.25%)	4 (14.81%)	
2	6 (18.75%)	4 (14.81%)	
3	1 (3.13%)	0 (0.00%)	
Weight loss, n (%)			0.248
0	25 (78.13%)	22 (81.48%)	
1	6 (18.75%)	2 (7.41%)	
2	1 (3.13%)	3 (11.11%)	
Anemia, n (%)			0.090
0	26 (81.25%)	19 (70.37%)	
1	2 (6.25%)	0 (0.00%)	
2	3 (9.38%)	2 (7.41%)	
3	1 (3.13%)	6 (22.22%)	
Nausea, n (%)			0.463
0	18 (56.25%)	20 (74.07%)	
1	6 (18.75%)	4 (14.81%)	
2	4 (12.50%)	2 (7.41%)	
3	4 (12.50%)	1 (3.70%)	
Gamma glutamyl transpeptidase, n (%)			0.089
0	27 (84.38%)	21 (77.78%)	
1	1 (3.13%)	5 (18.52%)	
2	4 (12.50%)	1 (3.70%)	
Aspartate aminotransferase level increased, n (%)			0.361
0	26 (81.25%)	22 (81.48%)	
1	4 (12.50%)	5 (18.52%)	
2	2 (6.25%)	0 (0.00%)	
Pruritus, n (%)			0.759
0	24 (75.00%)	18 (66.67%)	
1	4 (12.50%)	6 (22.22%)	
2	2 (6.25%)	2 (7.41%)	
3	2 (6.25%)	1 (3.70%)	
Leukopenia, n (%)			0.067
0	23 (71.88%)	17 (62.96%)	
1	8 (25.00%)	4 (14.81%)	
2	1 (3.13%)	6 (22.22%)	
Alanine aminotransferase level increased, n (%)			0.423
0	21 (65.63%)	21 (77.78%)	
1	7 (21.88%)	5 (18.52%)	
2	4 (12.50%)	1 (3.70%)	
Blood triglycerides increased, n (%)			1.000
0	29 (90.63%)	24 (88.89%)	
1	3 (9.38%)	3 (11.11%)	
Neutrophil count decreased, n (%)			0.255
0	27 (84.38%)	18 (66.67%)	
1	4 (12.50%)	8 (29.63%)	
2	1 (3.13%)	1 (3.70%)	
Blood glucose increased, n (%)			0.238
0	29 (90.63%)	20 (74.07%)	
1	2 (6.25%)	5 (18.52%)	
2	1 (3.13%)	2 (7.41%)	
Platelet count decreased, n (%)			0.379
0	24 (75.00%)	22 (81.48%)	
1	6 (18.75%)	4 (14.81%)	
2	0 (0%)	1 (3.70%)	
3	2 (6.25%)	0 (0.00%)	
Blood bilirubin increased, n (%)			0.275
0	27 (84.38%)	20 (74.07%)	
1	4 (12.50%)	3 (11.11%)	
2	1 (3.13%)	4 (14.81%)	
Erythra, n (%)			0.412
0	26 (81.25%)	23 (85.19%)	
1	4 (12.50%)	1 (3.70%)	
2	2 (6.25%)	3 (11.11%)	
Total AEs, n (%)			0.298
Grade 1–2	106 (86.88%)	103 (91.15%)	
Grade 3	16 (13.11%)	10 (8.85%)	

Because both arms received GC-based chemoradiation, attribution of specific adverse events to immunotherapy versus chemoradiation is tentative; immune-related events are therefore not presented as a separate category. AEs, adverse events; NACRT, neoadjuvant chemoradiotherapy; PD-1, programmed cell death protein-1.

## 4 Discussion

The present single-center analysis provides early but encouraging evidence that the addition of PD-1 blockade to gemcitabine–cisplatin-based NACRT can substantially improve tumor eradication in individuals with MIBC, without compromising peri-operative safety. Among 59 consecutive patients with cT2–T4aN0M0 disease who proceeded to RC at our institution between 2021 and 2024, the combination strategy delivered a PCRR of 71.88%, compared to 44.44% for NACRT alone—a difference that reached statistical significance despite the modest sample size (p=0.033). The overall PDR also numerically favored the combination regimen (87.50% *vs*. 74.07%), although the difference did not reach the conventional threshold for statistical significance, likely because NACRT alone already provides substantial cytoreduction (15). Importantly, no grade 4 toxicities or treatment-related deaths occurred, and the incidence of grade ≥ 3 AEs remained similar between groups (13.11% *vs*. 8.85%, p=0.298). Within the immunotherapy cohort, outcomes for patients receiving toripalimab and those receiving tislelizumab were comparable regarding complete response and downstaging, suggesting that the benefit represents a mechanism-class effect rather than molecule-specific activity (10, 16, 17).

The subgroup analysis suggested a higher PCRR in the overall cohort with NACRT plus PD−1 inhibition, driven primarily by an apparent benefit in the cT2 subgroup (94.44% *vs*. 50.00%, p=0.020), whereas PCRR and PDR rates were comparable between arms in cT3–cT4a disease. Given the retrospective design and limited sample size, this observation should be regarded as exploratory evidence of a potential therapeutic synergy between PD−1 blockade and NACRT in organ−confined MIBC. Such synergy might relate to lower tumor burden and more favorable tumor biology, which could enhance immunologic responsiveness (18). These findings should be interpreted cautiously and validated in future prospective, adequately powered studies.

Due to the limited availability of eligible patients within the predefined study period, a total of 59 participants were ultimately included: 32 in the NACRT plus PD−1 blockade group and 27 in the NACRT alone group. Although the actual sample size did not meet the pre−estimated requirement of 120 participants, thereby reducing statistical power, patient recruitment proved challenging. In China, the proportion of MIBC patients willing to undergo radical cystectomy remains low, and acceptance of neoadjuvant therapy is even lower. Consequently, each enrolled case represents a rare and valuable contribution to the study. It should also be noted that the NACRT plus PD−1 blockade group contained a numerically greater proportion of patients with clinical T2 disease compared to the NACRT alone group. Although this difference did not achieve statistical significance, such baseline imbalances are clinically relevant in relatively small cohorts, as cT2 tumors are typically associated with lower tumor burden, less extravesical extension, and higher likelihood of achieving pathological complete response. This stage distribution could therefore have contributed, at least in part, to the pCRR advantage observed for the combination regimen. Adjustment for clinical T stage in our multivariate logistic regression confirmed that stage remained an independent predictor of pCRR, but residual confounding cannot be entirely excluded, underscoring the need for prospective randomization with stage stratification in future studies.

This observation aligns with emerging evidence from recent neoadjuvant immunotherapy trials in MIBC, which have reported numerically higher pCR rates in patients with ≤cT2 stage disease compared to those with extravesical extension (19–21). While the small numbers in each subgroup preclude definitive conclusions, our results raise the hypothesis that baseline tumor stage could serve as a useful biomarker for selecting patients most likely to benefit from treatment intensification with PD−1 inhibitors in the neoadjuvant chemoradiation setting. Prospective randomized trials with balanced baseline staging and larger sample sizes are warranted to confirm this potential stage−dependent effect.

Taken together, despite potential stage-related influences, the collective data from our cohort indicate that the combination of PD−1 blockade with NACRT confers a meaningful pathological response advantage over NACRT alone, warranting contextualization against outcomes reported in historical and contemporary neoadjuvant studies. The PCRR achieved with NACRT alone in our series (44.44%) exceeds the 23.08% typically reported for cisplatin-based triplet chemotherapy delivered without radiation, confirming the intrinsic cytoreductive power of contemporary intensity-modulated pelvic irradiation when combined with radiosensitizing cytotoxic agents (10, 18, 22, 23). The addition of PD-1 inhibition further increased the PCRR to nearly three-quarters of treated individuals, surpassing the 31–42% rates reported for single-agent immune checkpoint blockade in the PURE-01 and ABACUS trials, and the 46–56% rates observed when pembrolizumab or nivolumab was combined with cisplatin–gemcitabine in BLASST-1 and HCRN-GU14-188 (24–27). These results position chemo-radio-immunotherapy as one of the most active neoadjuvant approaches evaluated to date. While cross−trial comparisons should be interpreted cautiously, relevant context can be drawn from the NEOBLADE trial (28), a double−blind, randomized phase II study assessing nintedanib, an oral angiokinase inhibitor, added to standard neoadjuvant gemcitabine–cisplatin in 120 patients with locally advanced MIBC. After a median follow−up of 33.5 months, pathological complete response (pCR) rates were 37% in the nintedanib arm versus 32% in the placebo arm (OR 1.25, 70% CI 0.84–1.87; p=0.28), with no survival benefit. Although overall grade ≥3 toxicity rates were similar, nintedanib significantly increased severe neutropenia (39% *vs*. 11%, p=0.0006) without improving pathological outcomes. Interpreted alongside our results, NEOBLADE demonstrates that simply intensifying systemic therapy in the neoadjuvant setting does not necessarily enhance tumor eradication and may add toxicity. In contrast, PD−1 blockade combined with NACRT in our cohort achieved a markedly higher pCRR (71.88%) than NACRT alone, without an increase in high−grade adverse events. This disparity may reflect the distinct biological mechanisms of angiogenesis inhibition versus immune checkpoint blockade, and the synergistic potential of chemoradiation with immunotherapy to promote tumor antigen release, dendritic cell activation, and sustained T−cell effector function. Collectively, these insights strengthen the rationale for prospective evaluation of chemo−radio−immunotherapy as a potentially more effective and tolerable intensification approach in MIBC compared with certain targeted−agent combinations, with convergent cytotoxic, radiation−induced, and immune−mediated effects offering biological plausibility for eradicating microscopic residual disease (29, 30).

Several non-mutually exclusive pathways could explain the apparent synergy (31). Ionizing radiation upregulates interferon-stimulated genes, increases major histocompatibility complex class I expression on tumor cells, and releases danger-associated molecular patterns such as calreticulin and high mobility group box 1, all of which facilitate dendritic cell priming of tumor-specific T lymphocytes (31–33). Gemcitabine augments this effect by preferentially depleting myeloid-derived suppressor cells, whereas cisplatin contributes by generating neoantigens through deoxyribonucleic acid (DNA) cross-link damage (34, 35). In an environment with intact PD-1 signaling, the influx of newly primed T cells becomes rapidly exhausted; however, pharmacologic blockade of PD-1 preserves effector cell function, converting local chemoradiation injury into a more durable systemic response (36, 37). Preclinical models have further demonstrated that fractionated radiation can broaden the T-cell receptor repertoire and facilitate epitope spreading, both of which may be critical for achieving sterilization of heterogeneous urothelial tumors (38, 39). Our decision to infuse the PD-1 antibody on day 8—after the first pulse of radiation and chemotherapy had initiated immunogenic cell death—might have provided an optimal temporal window for immune reinforcement, although this hypothesis warrants prospective pharmacodynamic validation.

Equally important is the observation that the intensified regimen did not translate into excess acute toxicity. The rates of peripheral neuropathy, myelosuppression, transaminase elevation, and dermatological reactions mirrored those observed with chemoradiation alone, and classic immune-related adverse events such as pneumonitis, colitis, or endocrinopathy remained infrequent. These findings aligned with early signals from phase I radio-immunotherapy platforms in rectal and head-and-neck cancers, which also reported acceptable safety profiles when PD-1/PD-L1 agents were intercalated with moderately hypofractionated fields (40, 41). From a surgical standpoint, avoidance of severe toxicity is crucial because delays in cystectomy can compromise oncological outcomes; the absence of treatment-related mortality or unexpected peri-operative morbidity in the present series reinforces the clinical feasibility of tri-modality therapy.

Despite these encouraging findings, several limitations warrant consideration. First, the retrospective design introduces inherent selection bias, although consecutive patient inclusion and comparable baseline demographics partially mitigate this concern. Second, while our cohort represents one of the largest reported for chemo−radio−immunotherapy in bladder cancer, it remains underpowered for robust assessment of certain secondary endpoints, such as specific toxicity subtypes or efficacy differences between PD−1 antibodies. While toripalimab and tislelizumab yielded comparable pathological responses in our analysis, the small subgroup sizes limit the ability to draw firm conclusions regarding differential efficacy. Considering their potential molecular and immunomodulatory distinctions, adequately powered prospective studies are warranted to clarify any clinically meaningful differences in the context of MIBC neoadjuvant chemo−radio−immunotherapy. Third, a numerical imbalance in baseline stage, with more cT2 cases in the NACRT plus PD−1 arm, may have influenced pathological complete response rates despite statistical adjustment; balanced stage distribution should be ensured in future randomized trials. Fourth, the median follow-up was insufficient to assess event-free or overall survival, outcomes crucial for determining long-term benefit; surveillance is ongoing. Fifth, attribution of adverse events to individual treatment components was not possible given the shared gemcitabine–cisplatin chemotherapy and pelvic radiotherapy in both arms, with PD−1 blockade only in the experimental arm. Potential immune-related events (e.g., hypothyroidism, rash) occurred concurrently with chemoradiation toxicities, precluding precise categorization; a dedicated PD−1 monotherapy cohort would be required to define the irAE spectrum. Sixth, histological grade was determined from initial TURBT specimens, which may underestimate the highest grade owing to sampling limitations. Post−cystectomy specimens, obtained after neoadjuvant therapy, could not retrospectively confirm initial grading or exclude concurrent high-grade disease. Seventh, all treatments were delivered at a single tertiary Chinese center, which may limit generalizability to other populations or healthcare settings. The observation of initial low-grade histology with confirmed muscle invasion is unusual, potentially reflecting tumor heterogeneity, sampling bias, or rare low-grade MIBC variants, and warrants further study. Finally, given the modest sample size and multiple pairwise comparisons, all statistical findings should be interpreted as exploratory and hypothesis−generating. To address these constraints, a multicenter randomized phase II trial is planned to compare NACRT with or without PD−1 blockade, using event-free survival as the primary endpoint, alongside comprehensive translational studies exploring immune contexture, T-cell clonality, and DNA damage response pathways in paired pre− and post−treatment specimens.

In summary, NACRT combined with PD-1 blockade demonstrated a promising advantage in enhancing pathological response in MIBC without introducing appreciable additional toxicity. However, as no survival data are yet available from this cohort, and given that pCRR is a surrogate endpoint of unproven predictive validity in the NACRT + ICI setting, the long-term clinical impact of our findings remains uncertain. A prospective, single-arm, multicenter phase II trial is underway to determine whether these short-term pathological responses translate into durable improvements in recurrence-free and overall survival, thereby establishing the prognostic value of pCRR in this therapeutic context.

## Data Availability

The datasets presented in this study can be found in online repositories. The names of the repository/repositories and accession number(s) can be found below: https://figshare.com/s/eace6bef23b6bb579c76.
